# Implementing the family-centered care model, parents’ satisfaction and experiences in neonatology

**DOI:** 10.1186/1824-7288-40-S2-A47

**Published:** 2014-10-09

**Authors:** Immacolata Dall’Oglio, Anna Portanova, Martina Fiori, Orsola Gawronski, Roberta Fida, Antonello Cocchieri, Gennaro Rocco, Emanuela Tiozzo, Jos M Latour

**Affiliations:** 1 Professional Development, Continuing Education and Nursing Research Service-Medical Direction, Bambino Gesù Children’s Hospital, IRCCS, Rome, Italy; 2 Department of Medical and Surgical Neonatology, Bambino Gesù Children’s Hospital, IRCCS, Rome, Italy; 3 W&O Psychology, Department of Psychology, Sapienza University of Rome, Italy; 4 Catholic University of Rome, Italy; 5 Centre of Excellence for Nursing Scholarship, Ipasvi Rome Nursing College; 6 School of Nursing, Plymouth University, Plymouth, UK

## Background

The quality of family-centered care (FCC) in Neonatal Intensive Care Unit (NICU) is often assessed through Parental satisfaction (PS). Empathic-N, a validated questionnaire to evaluate PS in NICU, was recently developed in the Netherlands [1].To our knowledge similar instruments have not yet been used in Italy. The aim of this project is to translate the 57-item Empathic-N questionnaire and to develop and adapted Italian version for post-NICU (Empathic-SN) taking the Italian cultural adaptation into account, and to test their psychometric validity.

## Materials and methods

The translation process followed a structured method including forward and backward translation [2].

The psychometric validation of both the Empathic-N and the Empathic-SN questionnaires is being performed by administering the questionnaires to parents of newborns discharged from 9 NICUs and post-NICUs across Italy.

Ethical approval is granted by the Bambino Gesù Children's Hospital. Written informed consent forms are collected.

## Results

150 questionnaires from NICU and 150 from post-NICU are being collected. Preliminary analyses showed a positive correlation between the questionnaire items and the overall satisfaction indicators. Reached scores ranged from 4.1 to 5.9 for the Empathic-N and from 4.2 to 5.7 for the Empathic-SN, on a 1-to-6 Likert scale. Results from Cronbach’s alpha coefficients attested the reliability of the scale. Thematic analysis of the open answers identified 385 quotations, coded into seven major themes, expressing parents’ experiences. Generally, a good overall parents’ satisfaction is showed. Further descriptive statistical analysis will be performed on the complete sample.

## Conclusion

Validity and reliability of the Italian version of the questionnaires assessed by psychometric testing is expected. The Empathic-N and Empathic-SN questionnaires, or their further versions, would constitute important tools to assess the actual quality of FCC in Italian NICUs and post-NICUs and to set the baseline for improvement interventions.
